# The impact of COVID-19 lockdown on physical activity and weight gain among active adult population in Israel: a cross-sectional study

**DOI:** 10.1186/s12889-021-11523-z

**Published:** 2021-08-06

**Authors:** Horesh Dor-Haim, Sara Katzburg, Polla Revach, Hagai Levine, Sharon Barak

**Affiliations:** 1 O2 Health Promotion and Sports Medicine Department, The Howard and Mary Edith Cosell Association for Physical Education, the Safra sports center, Hebrew University, Givat Ram, Jerusalem, Israel; 2 Department of Developmental Biology and Cancer Research, Israel-Canada Medical Research Institute, Faculty of Medicine, Hebrew University of Jerusalem, Jerusalem, Israel; 3 Braun School of Public Health and Community Medicine, Hebrew University of Jerusalem, Jerusalem, Israel; 4 The Edmond and Lily Safra Children’s Hospital, Department of Pediatric Rehabilitation, The Chaim Sheba Medical Center, Ramat-Gan, Israel; 5 Kaye Academic College of Education, Beer-Sheva, Israel; 6 College of Public Health Ben-Gurion University of the Negev, Beer-Sheva, Israel

**Keywords:** Physical activity, Exercise, Health status, Weight, Digital fitness modalities, Fitness applications

## Abstract

**Background:**

The COVID-19 outbreak holds public health concerns. The stay-at-home increases sedentary behavior, with unintended adverse outcomes. Since organized recreation and sports facilities were closed, we aimed to study how the crisis of closure affected exercise habits and weight gain among the trainee population in Israel. We examined differences in weight gain among individuals with different PA activities and assessed their ability to adapt to digital media as an alternative training structure.

**Methods:**

A cross-sectional survey consisted of a multiple-choice questionnaire obtained using a web-based survey application. Trainees (1202) who exercised steadily anonymously answered the questionnaire sent by their coaches regarding their activity and weight gain during lockdown times.

**Results:**

Results confirmed that 70% of Israelis trained less than their usual routine, 60% used digital media for training, 55% gained weight. Half of the respondents gained more than 2 kg, with an average increase of 1.2 kg. However, those who exhibited a higher physical activity level gained less weight. Using digital media for training was associated with higher physical activity levels. The aged population was less likely to use digital media.

**Conclusions:**

Since increased sedentary behavior could increase the risk for potential worsening of health conditions, health agencies should look for strategies, including digital remote media training to promote physical activity and subsequently, preventing the increased burden of future comorbidities worsening by a sedentary lifestyle.

Approval: by the Helsinki ethics committee of Sheba Medical Center (6504–19-SMC).

**Supplementary Information:**

The online version contains supplementary material available at 10.1186/s12889-021-11523-z.

## Background

The coronavirus disease 2019 (COVID-19) has an impact on physical activity (PA) behaviors worldwide. People around the world stayed at home self-isolated due to the lockdown policy [1]. Although the lockdown is essential and is the best recommendation for preventing the spread of the disease, it may create a new challenge. Staying at home for a prolonged period can lead to disturbing consequences such as weight gain, social isolation [2] and may also cause a reduction in PA levels [2, 3]. The decrease in PA level may be especially apparent among active individuals habitually practicing sports. Diminished PA resulting from home isolation may worsen a wide range of health conditions, including the chronic ones, such as cardiac and metabolic diseases [2–5] as well as infectious diseases, due to negative immune-modulation [6–11] even without substantial weight gain [12, 13]. Therefore, maintaining an active lifestyle at home including mainly PA, is extremely important for the general populations’ health, especially for people with additional risk factors including old adults during the quarantine [14–16].

According to the recommendation of the World Health Organization (WHO), the American College of Sports Medicine (ACSM) Position Stand, and PA guidelines for healthy adults, adults should do at least 150 min of moderate aerobic exercise or do at least 75 min of vigorous aerobic exercise during the week [16, 17]. This is in addition to muscle-strengthening activities, involving major muscle groups, for two or more days weekly [16, 18]. According to the Israeli National Health Interview Survey – INHIS 2013–2015 [19] only one-third of the Israeli population aged 21 and over complies with the WHOs’ recommendations and walking being the sport of choice for most people [19]. As the consequence of physical inactivity is rising globally, WHO had developed a new Global Action Plan to promote PA in 2018–2030 [17]. However, with COVID-19 striking globally, adherence and devotion to PA is much more challenging, even for active individuals [20].

Longitudinal observational studies and experimental data identified determinants with strong causal associations to adherence to PA, such as characteristics of the person and their lifestyle habits, features of environments, and type of the activity itself [21, 22]. Markedly, socioeconomic status, education level, living arrangements, health status, physical fitness, and depression are factors that affect adherence, especially in old adults [23–25]. Improving adherence to PA, at times pandemics, can have a significant impact on longevity, quality of life, and health care costs in the long run [23]. Thus, it is essential to understand how the lockdown policy has affected PA behavior. Despite the barriers, it is crucial to maintain the regular regime of PA practiced on pre-pandemic days, to keep good mental and physical health [2]. Hence, it is worthwhile to study how to develop methods and strategies that allow remote PA among the regularly trained population.

Recent advances in mobile technology enable novel approaches to PA exercises using remote guidance and support. There is a wide range of at-home programs (video- or app-guided equipment-free), for aerobics or strength training. Studying the feasibility of at-home, web-based, interactive exercise programs [25] could enable researchers and health organizations to plan better strategies to encourage participants to engage in their PA.

We hypothesized that quarantine and closure of organized exercise installations would impair trainees’ exercise and habits, alter the daily caloric balance and lead to weight gain. Studies around the world showed a negative impact on physical activity during COVID-19 closure [26–29]. Therefore, the current study aimed to study the effects of lockdown policy on Israeli trainees’ PA level and exercising habits, analyze the association between these habits and weight gain, and evaluate the adaptability of the trainees to home-based digital technologies to maintain a healthy lifestyle during COVID-19 pandemics.

## Methods

The cross-sectional survey (convenience sampling) examined the PA of participants (n-1202) during March–April 2020 (May 20 to May 10). participants answered anonymously a questionnaire that consisted of multiple-choice questions (ST1). The questionnaire was developed for the study and was not used or published before. The questionnaire included information relating to demographics and exercise habits, before and during quarantine, medical status (ST1). Determination of the sample size done by an a priori power analysis showing that for multiple regression analysis (F test) with small effect size (f2 = 0.02), α err probability = 0.05, and maximum four predictors, the total sample size required for achieving actual power of 0.80 is *n* = 602. For one-way analysis of variance (F test), with a small effect size (f = 0.10), α err probability = 0.05, and five groups, the total sample size required for achieving actual power of 0.80 is *n* = 1200, using G*Power 3.0.10 for Power analysis calculations. The survey was obtained using the IMKFORMS, conducted web-based survey application.

The IMKFORMS system is optimized for mobile phones, collecting information from the respondents and export it into an Excel file (ST2). The survey was delivered via WhatsApp groups of trainers, targeting their trainees who were exercising in classes, gyms, and community centers. The trainers had no access to the database. Approval for the study by the Helsinki ethics committee of Sheba Medical Center (6504–19-SMC). All participants gave written informed consent.

### Data analysis

Description of demographics, PA, and training characteristics, were calculated using descriptive statistics (mean, median, range, and 95% confidence of interval) and chi-squared tests (ST1). Examination of the associations between age, PA level, and health status factors was done using Spearman rank correlations. Based on weight gain status participants, were divided into two groups (no weight gain vs. weight gain). Between-group differences in PA frequency were compared, by using the Mann-Whitney U test. For the evaluation of PA frequency, participants were subdivided into four groups according to their weekly PA frequency (0, 1, 2, 3 days /week (. Between-group differences in health status were done using the Mann-Whitney U test. Participant’s intention to return to the previous PA level, based on their health status, was compared using the Kruskal-Wallis test and Conover posthoc test. Evaluation of age differences among individuals within each PA frequency group was done using the one-way analysis of variance with Tukey-Kramer post-test for all pairwise comparisons was done. For factors predicting PA frequency and weight gain, “two forward stepwise multiple linear regression” was done (ST3-ST4). Only variables having significant correlations with the dependent variables and matched the study’s hypothesis were included [ST2, ST3). For multicollinearity, independent variables were examined using the variance of inflation factor > 10 [30]. The alpha level for inclusion criteria was 0.05, the alpha level for exclusion was 0.10. The coefficient of reliability (or consistency), of the questionnaire, was measured using Cronbach’s alpha [31]. Using Little’s MCAR test showed that data were not missing “at random”. However, to avoid biased parameter estimates, a complete case analysis [32], was done and individual missing data were excluded.

## Results

1202 adults participated in the study. Age range: 18–85 years (48.52 + 15.60 Old adults defined as > 65 years old, (*n* = 227). 75.0% (901) females and 25% (301) males (chi-squared = 299.50, *p* <  0.001). The questionnaire’s Cronbach’s was acceptable (Cronbach alpha = 0.70).

### Physical activity and exercising characteristics

Twenty-five percent of participants exercised in the fitness room 3.49% exercised with a personal coach, participating in more than one organized activity (26.03%), or not (8.48%). About one-third exercised as part of a team or in organized PA groups (36.52% of the sample; chi-squared = 439.75, *p* <  0.01). Seventy percent reported that they exercised less than usual, while 63% (less active 22.46%; significantly less active 48.08%, chi-squared = 355.15, *p* < 0.001), Table 1) reported that in the past month they exercised three times a week or less. Half of the participants (51.50%) reported that they intend to return to PA activities as soon as the restrictions end or when PA will be allowed by the regulations (35.90%). Only 0.20% of the participants informed about health status in the past months and therefore have no intention of returning to their previous PA types. 6.30% reported they were afraid to return to their previous PA types, and 6.10% have not decided whether or not to return to their previous PA regimes (chi-squared = 736.10, *p* < 0.001).

**Table 1:** Physical activity characteristics

Physical activity characteristic		n	%
Organized physical activity* (*n* = 1202)	In a team or an organized group	439	36.52%
	Fitness room	306	25.45%
	Personal training	42	3.49%
	More than one organized activity	313	26.03%
	Not in an organized activity	102	8.48%
Current physical activity level vs. before Corona* (n = 1202)	Similar level	185	15.39%
	Less active	270	22.46%
	Significantly less active	578	48.08%
	More active	169	14.05%
Home digital physical activity modalities* (n = 1202)	Not used	265	22.04%
	I tried but it wasn’t good for me	256	21.29%
	Movies in the internet/TV	196	16.30%
	Programs like Zoom	285	23.71%
	Training applications	54	4.49%
	Several digital training modalities	146	12.14%
Intention to return to previous physical activity* types (*n* = 1198)	No – afraid of the virus	75	6.30%
	Not decided	73	6.10%
	Yes, considering activity will be in accordance with the regulations	430	35.90%
	Yes, immediately	618	51.50%
	No, my health deteriorated in the last month	2	0.20%
Physical activity frequency* (n = 1202)	0 / week	113	9.40%
	1 / week	172	14.30%
	2/ week	225	18.71%
	3/ week	245	20.38%
	4 / week or more	447	37.18%

Notes: sample size (n) and prevalence (%); * significant differences in prevalence at the p < 0.001 (Chi-squared,2-tailed)

37.18% of the participants reported exercising 4 times/week as compared to 20.38% (3 times/week), 18.71% (2 times/week), 14.30% (1 day a week) and 9.40% who did not practice at all (chi-squared = 262.23, *p* < 0.01). The median physical activity was 3 times/week (Table 1)

### Health status of the participants

Overall, 87.3% reported to be in good health, 11.3% reported having good health in conjunction with chronic diseases, and 1.3% were suffering from chronic diseases and disability (chi-squared = 1614.12, *p* < 0.001). Regarding weight gain, 45.2% did not gain weight, 16.2% gained 1Kg, 18.8% gained 2KG, 11.2% gained 3Kg, 8.6% gained 4 Kg or more (chi-squared = 1181.14, p < 0.001). The median weight gain was 1 kg (Table 2).

**Table 2:** Associations between age, physical activity, and health parameters

		Age	Health Status	Weight gain	physical activity frequency	Home digital physical activity modalities
Age	r	–	0.229	−0.080	0.094	−0.033
	p	–	< 0.001*	0.006*	0.001*	0.258
	n	–	1175	1178	1175	1178
Health status	r	–	–	− 0.002	− 0.031	− 0.052
	p	–	–	0.952	0.283	0.072
	n	–	–	1202	1202	1202
Weight gain	r	–	–	–	−0.382	− 0.111
	p	–	–	–	< 0.001*	< 0.001*
	n	–	–	–	1202	1202
physical activity frequency	r	–	–	–	–	0.295
	p	–	–	–	–	< 0.001*
	n	–	–	–	–	1202

Notes: r- Spearman rank correlation coefficients; * p - *p* < 0.05 level (2-tailed), n- sample size

### Associations between age, physical activity, and health parameters

Significant positive correlations between age and health status were observed (r = 0.229, *p* < 0.0001). Significant positive correlations between Weight gain and PA frequency (r = − 0.38, *p* < 0.001, r ranges from − 0.080 to 0.229; p < 0.05) and usage of home digital physical activity modalities (r = − 0.111, p < 0.001) was noticed. PA frequency was significantly positive correlated with usage of home digital PA modalities (r = 0.295, p < 0.001; Table 2).

**Fig. 1:**
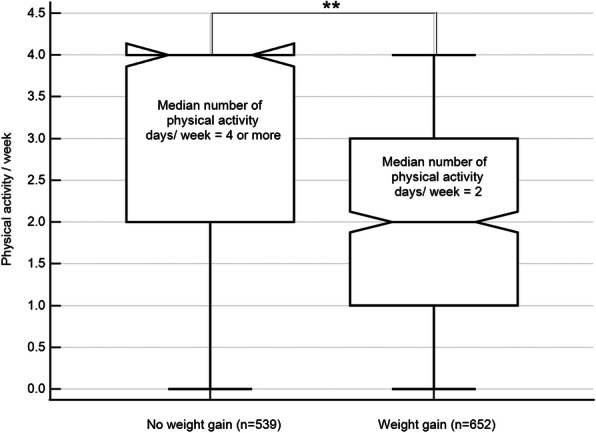
Physical activity level based on weight gain group. ** Significant between-group differences at *p* < 0.01 (Mann-Whitney U test = 109,800.00); The central box represents the values from the lower to upper quartile (25 to 75 percentile); the vertical line extends from the minimum to the maximum value; the middle line represents the median

In comparison to participants gaining weight, participants who did not gain weight were significantly more physically active (*P* < 0.001, the median number of PA days = 2 vs. 4, respectively; Fig. 1).

**Fig. 2:**
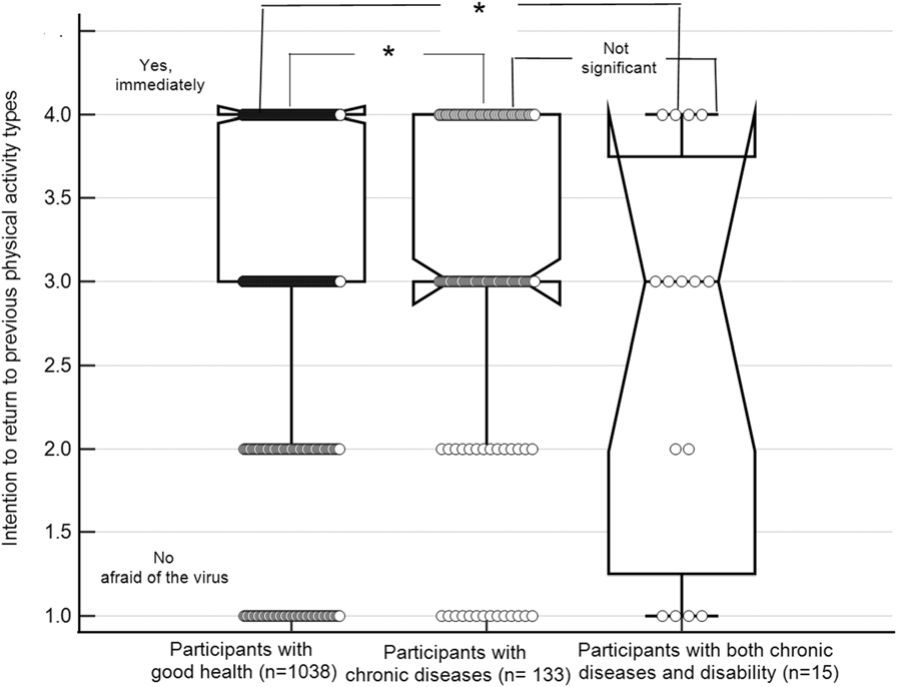
Return to physical activity intentions based on health status. Significant at *p* < 0.05 (Conover post-hoc test); Intention to return to physical activity category 5 (− no, because my health deteriorated), was omitted from the analysis as it included only two participants; the central box represents the values from the lower to upper quartile (25 to 75 percentile); the vertical line extends from the minimum to the maximum value, excluding outside values which are displayed as separate points. An outside value is defined as a value that is smaller than the lower quartile minus 1.5 times the interquartile range, or larger than the upper quartile plus 1.5 times the interquartile range; the middle line represents the median

Compared to individuals with chronic diseases or chronic diseases with disabilities, individuals with good health had a significantly higher intention to immediately return to their previous PA level (P < 0.001, Fig. 2)

**Fig. 3:**
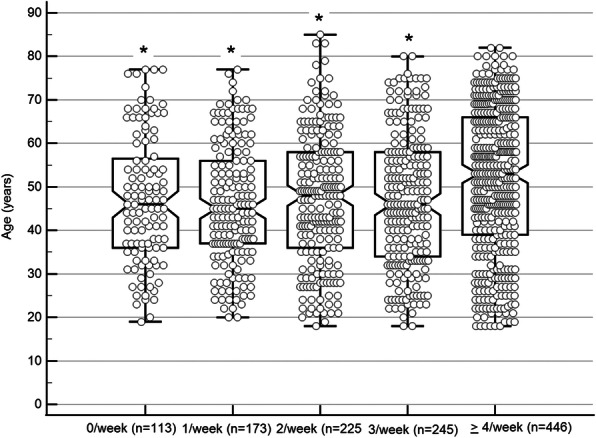
Physical activity frequency - age differences. * significantly different than 4/week group at p < 0.05 (Tukey-Kramer test for all pairwise comparisons); the central box represents the values from the lower to upper quartile (25 to 75 percentile); the vertical line extends from the minimum to the maximum value, excluding outside values which are displayed as separate points. An outside value is defined as a value that is smaller than the lower quartile minus 1.5 times the interquartile range, or larger than the upper quartile plus 1.5 times the interquartile range; the middle line represents the median

No age-related differences were observed in the different categories to return to previous PA types. Similarly, no significant between-group differences were found, in the various health status groups with PA frequency (Fig. 3)

Participants who conducted PA > 4/week (*n* = 446), were significantly older than participants who practiced PA less than 4/week (*n* = 756; F = 4.14, *p* ≤ 0.002; Fig. 3). However, comparing the participants who were physically active (conducting PA > 3/week), there were no significant between-group differences concerning their health status (good health, 61.90%; good health with chronic diseases, 65.69%; and chronic diseases with a disability, 62.50%). Multiple regression analysis showed that older age and the greater usage of home physical activity modalities predicted greater frequency of PA level (adjusted R2 = 0.10; F ratio = 69.51; *p* < 0.001). However, only reduced PA frequency significantly predicted weight gain (adjusted R2 = 0.156; F ratio = 71.05; p < 0.001). For additional information (Table 3).

**Table 3:** Prediction of physical activity level and weight

Dependent variables	Independent variables	B Coefficient	Standard error	t	p
Last month physical activity frequency	(Constant)	1.40			
	Age	0.007	0.002	3.42	< 0.001
	Usage of home digital physical activity modalities	0.26	0.022	11.36	< 0.001
	**R**^**2**^ = 0.10; **Adjusted R**^**2**^ = 0.10; **F ratio** = 69.51; ***P*** < 0.001				
Weight gain	(Constant)	2.469			
	Age	−0.004	0.002	−1.949	0.051
	Physical activity frequency	−0.385	0.028	−13.357	< 0.001
	Usage of home digital physical activity modalities	−0.008	0.024	−0.353	0.723
	**R**^2^ = 0.158; **Adjusted R**^**2**^ = 0.156; **F ratio** = 71.05; ***P*** < 0.0001				

Notes: continuous variables: included are only variables that had significant correlations with the dependent variables. Categorical variables: only variables that differed significantly in the dependent variables, were included; the Variance inflation factor in all analyses was < 10

## Discussion

Our study shows that PA level significantly decreased during the COVID-19 lockdown. Of the participants, 70% reported that they exercised less than usual, while 63% (Table 1) reported exercising 3 times a week or less, and correlated to increased weight gain (54.8%). PA level correlated with the usage of home digital PA modalities (Tables 2 and 3).

The current study reveals the negative effect of lockdown and social distancing policy on PA level and exercising habits and health status among physically active adults during COVID-19. We assume that the closure resulted in a reduction in PA below the minimum recommendations of health organizations which is in line with other studies that showed a decline in PA level during closure times [2, 9, 15, 26, 27, 29]. The study suggests that pandemics such as the current pandemic [1], pose unique health issues caused by the requirements to stay at home, leading to a reduction in PA, among all people and especially among physically active individuals habitually practicing sports [33]. Although lockdown may be essential to mitigate the pandemic, it also generates new health challenges. Staying at home for a prolonged time may increase sedentary habit [2, 3] and decrease PA level [12, 34] that may lead to disturbing consequences such as an increased risk of worsening health conditions (including chronic ones) [2, 3], weight gain [12, 34] insufficient sunlight exposure, social isolation [2] and can affect metabolic health [35]. While regular exercise has a positive impact on mental health and overall mood [36], prolonged stay at home can lead to inactivity and contribute to anxiety and depression, which leads to an even more sedentary lifestyle and weight gain [9, 26, 37–39]. Altogether, sedentary behavior and low levels of physical activity can have adverse effects on well-being and quality of life.

The Israeli Ministry of Health closed all organized activities in mid-March, including gyms and fitness centers [40]. The Government enforced restrictions upon outdoor and recreational activities and limited outdoor and social activities to a maximal radius of 100 m distant from home. Whereas the blockade policy aimed to prevent the spread of COVID-19, there was no reference or guidance on maintaining normal PA levels during times of social distancing and isolation, regardless of the health implications that could result from physical inactivity. Thus, our survey exhibited the difficulty of maintaining a healthy and active lifestyle during the lockdown. Seventy percent of the participants, who were physically active in their daily life before the pandemic broke, indicated a significant decrease in the PA level related to increased weight gain. A correlation between age, health status, and the reduced PA level was found. The population with poor health status was the most prone to pandemic quarantine. They were less likely to initiate home-based activities. Their health toll is higher as a sequel of physical inactivity and can lead to deterioration of their health condition. In the old-adult population, there was a positive association between age and the frequency of physical activity. They were less likely to engage in digital home-based physical activity but had proficient and regular training habits that did not change even in time of closure. This surprising result may reflect the relative resilience of the aged healthy trained population, striving not to neglect their lifestyle habits. For the less active older adult population, the situation is different and should be further studied. Cardiovascular deconditioning might increase the risk for cardiovascular disease as deconditioning of the musculoskeletal system might increase frailty, Osteoporosis, and risk of falling among the elderly [41, 42].

Physical inactivity and a sedentary lifestyle are risk factors for coronary vascular disease, diabetes, and many more non-communicable diseases (NCDs). Although the general activity of the old adult group is limited, a priory, nevertheless, this population is the most vulnerable to high morbidity caused by COVID-19 [43–45]. Up to 40 million individuals die each year from NCDs, 20–30% of these cases related to a deficiency in PA [46]. The total volume of accumulated sedentary time in prolonged, uninterrupted bouts is also associated with all-causes of mortality. Therefore, any PA guidelines should aim to reduce the sedentary time to avoid the risk of death [47, 48].

Lifestyles and PA have been changed globally during the coronavirus pandemic lockdown [2, 33, 49]. Data collected by Fitbit wristwatches from over 30 million active users around the world showed a decrease in PA of 38% in Spain, a 14% decrease in North America, and a 24% decrease in Israel [50]. Garmin users reported a massive decline in the overall number of steps taken during the second 2 weeks of March [51]. Therefore, it is essential to grant access to activity substitutes, especially for old adults, to keep them fit and healthy at their own homes. However, the ability of people to change to a new, unaccustomed regime that uses digital technology and social media is limited. It is necessary to make digital technology effective and accessible for all people, particularly for those with special needs. Furthermore, there is a need to implement and improve digital technology to enable a better attitude toward digital technology. Such technologies can help introduce a broader range of PA and media solutions, focusing on different populations, communities, languages, and cultures. By utilizing digital technology, it is possible to deliver content designed for remote PA exercise at home when regular training options are unavailable.

The current survey found that 55% of the participants gained weight during the March–April pandemic quarantine. Weight gain was significantly correlated negatively with age, PA level, and use of home digital PA modalities. However, a multiple regression analysis showed that only a reduction in PA level significantly predicted weight gain. Weight gain and metabolic syndrome are a global crucial public health issue concerning sedentary behavior, during the recent COVID-19 pandemic [52, 53]. The syndrome is related to increased weight gain, abdominal obesity, hypertriglyceridemia, low high-density lipoprotein cholesterol, high blood pressure, and hyperglycemia [52, 54]. People having metabolic syndrome are at increased risk of developing diabetes and cardiovascular disease [54]. According to the Israeli Central Bureau of Statistics, 48% of adults are overweight [55]. The study surveyed overall active individuals (57% of study participants were performing PA at least three times a week), with a considerable average increase in weight (1.25 kg). However, participants who conducted PA > 4 times/week did not gain weight. The weighted mean weight regained with or without exercise training was only 0.28 and 0.33 kg/month, respectively. Based on observational studies, an actual increase in energy expenditure of PA of approximately 6300–8400 kJ/week (1500–2000 kcal/week) is associated with improved weight maintenance [56]. The increased weight gain outcome in the active individuals, adherence is explained by the decrease in non-exercise activity thermogenesis (NEAT) that is the energy expenditure of all physical activities other than volitional sporting-like exercise. Since activity levels declined, it is not surprising that the whole NEAT decreased substantially, reflecting the increased indoor inactivity time. To bring it back to the former state there is a need to develop strategies by re-engineering our home environments [57]. Exercise is also associated with long-term weight loss through the relationship of its associated psychological changes, together with improved nutrition, than through direct effects of energy expenditures, which are typically minimal in deconditioned individuals. There is a mutual relationship between exercise and diet [58–60]. Exercise can induce changes in mood, body image, self-efficacy, self-esteem and improves eating habits and weight loss [58, 60]. Likewise, diet and dietary practices affect mental well-being [59] Engaging in healthy behaviors leads to increased motivation for the enhancement of well-being and improvement of mental health.

That mutual effect has a better impact than the direct effects of energy expenditures that are typically minimal in deconditioned individuals [13, 61, 62]. Weight gain is associated not only with PA level but also with nutrition. COVID-19 has influenced people eating habits. Preliminary data from the Brookdale Institute survey in Israel showed changes in nutritional routine during the home lockdown, wherein people were reporting consuming more food, more sweets, and snacks [35].

The current survey showed PA behavioral changes among the trainee population. Even though participants have had more spare time to exercise, they had numerous difficulties maintaining their PA routine. This effect of the pandemic health crisis on a person’s well-trained behavior is paradoxical. Hypothetically, a person who routinely exercises has acquired a “toolbox” filled with skills and training habits. Naturally, we would expect him/her to preserve and use his/her skills while in urgent need. Paradoxically the study shows that most participants reduced their PA level, and only 15% increased their PA. One explanation for this aforementioned paradoxical effect might be related to stress. More specifically, the stressful situation caused by the pandemic might have brought difficulties in maintaining an active lifestyle. Another possible explanation for the observed reduction in the PA level might be related to differences in adherence to individualized and group exercise. At times of closure, there is no option to use the existing infrastructure for PA in residential areas such as parks and fitness clubs as Israel had closed all recreation and sports facilities. Therefore, the ability to exercise with others was limited. Exercising with others was associated with superior adherence behavior [63]. Adults exposed to a team-building activity intervention reported greater adherence than those who participated in a standard-care program [64]. An alternative to the group activity might be engaging in PA with digital technology. Appropriate adaptation to the changed situation allowed participants to maintain their active routine even during the lockdown.

However, that adjustment was age-dependent, and older adults found it hard to adopt digital technology as an alternative to organized PA [65, 66]. For an active adult population, the consequences of inactivity and improper diet will be visible long after the crisis is over. It may result in an increased burden of NCDs, increased risk for morbidity due to metabolic, cardiovascular disease, and muscular deconditioning. Thus, the various governmental institutions and health agencies need to consider how to prevent it. An emergency plan must be built, within the health promotion actions to include specific policies and guidelines for home-based physical training. We hence encourage the various governmental institutions and health organizations to consist clear instructions for PA in nationwide lockdowns, allowing a safe performance of outdoor PAs (walking, running, or other individual sports) and thus prevent any future pandemic from generating unfavorable consequences that should arise due to acute cessation of physical activity. Furthermore, it might be advisable to engage in community-wide campaigns that deliver messages regarding upholding the PA level during lockdown by using the media such as television, radio, and newspaper columns. The surveyed group was different from the general population. This group was used to train regularly, acquired good healthy habits before the covid-19 pandemic, and thus were more motivated to adhere. Presumably, the general population was even worse at the time of the closure. This study was subject to several methodological limitations. The majority of the participants who responded to the survey were women, the generalizability of this study may be limited mainly to overall active females. Participants had to report the number of days per week in which they practiced PA > 20 min rather than depict the actual time and intensity. Therefore, there is no certainty as to whether they followed the WHO guidelines. Besides, this cross-sectional study refers to reports on PA level and weight. Future studies should incorporate objective measures (e.g., accelerometry and weight measurements). Finally, the data presented in this study represents a snapshot of the current situation relevant to the early stages of the pandemic in Israel. As the pandemic evolves and subsequent national and institutional new policies and practices developed and implemented, future follow-up investigations will be necessary to understand the effects of COVID-19 over time.

## Conclusion

In conclusion, this study showed a negative effect of lockdown and social distancing policy on PA levels and weight gain. However, those who exhibited higher PA levels gained less weight. Using digital media for training was associated with a higher PA level. Therefore, there is a need for governmental and health agencies to rethink a health policy in which PA will be more accessible during pandemic crises, especially for the aged population and people with special physical and medical needs. Outdoor or home-based remote training using Digital technology can be a suitable substitute for gym-based installations. 51% of survey participants, including aged and those with medical risk factors, reported their intention to go back to their original organized activity. About 10% were concerned about returning to their pre-pandemic exercise regime. These concerns illustrate the importance of organized PA in peoples’ lives as an essential need for their physical, social, and mental health. All this should be taken into consideration when organized activity is closed.

## Supplementary Information

**Additional file 1.** ST1 The multiple-choice questionnaires.

**Additional file 2.** ST2 Raw collecting data.

**Additional file 3.** ST3 Regression physical activity level.

**Additional file 4.** ST4 Regression weight gain.

## Data Availability

Is included as supplements: ST1-ST4.

## Abbreviations

PA: physical activity
ACSM: American College of Sports Medicine
WHO: World Health Organization
NCD’s: non-communicable diseases
