# Supplementary material for: The impact of COVID-19 lockdown on physical activity and weight gain among active adult population in Israel: a cross-sectional study
Source: BMC Public Health. 2021 Aug 6;21:1521. doi: 10.1186/s12889-021-11523-z (PMC8343341; doi:10.1186/s12889-021-11523-z)
Supplement: Supplementary file 1 — Additional file 1. ST1 The multiple-choice questionnaires. [file 12889_2021_11523_MOESM1_ESM.docx]

**The forum for Public Health, O2 institute for Health Promotion and Sports Medicine at ADAM wing, cosell center for physical education, Hebrew University**

**How old are you**?

**Gender**

male

female

**Regarding regular activities in gyms, classes, personal or group training**

**Do you exercise regularly?**

 Not as part of an organized group

 Yes, as part of a gym

 Yes, in a group, in a private class or in the community

 Yes, personal training

 Yes, in more than one framework

**What is your health condition?**

 Normal good health

 I feel good, but suffer from background illnesses and risk factors

 I have background illnesses that limit my daily functioning

**weight gain**

**In your estimation how many kg did you gain over the past months**

no gain

1Kg

2 Kg

3 Kg

4 Kg and more

**For training you have done proactively for over 20 minutes**

**How often did you exercise last month?**

 I do not exercise at all

 Exercise once a week

 Exercise twice a week regularly

 Exercise 3 times a week regularly

 Exercise 4 times a week regularly and more

**Regarding your general activity and initiated physical exercise**

**Are you less active than you were before Corona?**

 Significantly less active

 A little less active

 Actively similar to what I was before

 More active than I was before

**Use of the Internet, apps or zoom software**

**Have you used digital means for home training?**

 Did not used

 I tried but it didn't suit me

 I use training apps

 I use videos for training that I find on the Internet or TV

 I use zoom or similar online training software.

**Assuming you are engaged in physical activity as part of a class, gym, personal training or group**

**Do you intend to return to activity and therapy at the same framework you practiced before?**

 Yes, immediately

 Yes, if conditions comply with the instructions of the Ministry of Health

 No, because I'm afraid to get infected with the Corona virus

 No, because my health deteriorated in the last month

 Haven't decided yet
