# Supplementary material for: The impact of COVID-19 lockdown on physical activity and weight gain among active adult population in Israel: a cross-sectional study
Source: BMC Public Health. 2021 Aug 6;21:1521. doi: 10.1186/s12889-021-11523-z (PMC8343341; doi:10.1186/s12889-021-11523-z)
Supplement: Supplementary file 3 — Additional file 3. ST3 Regression physical activity level. [file 12889_2021_11523_MOESM3_ESM.docx]

**Regression physical activity level**

**Regression**

| **Notes** | | |
| --- | --- | --- |
| Input | Data | D:\SharonDocuments\Desktop\Corona and PA.sav |
|  | Active Dataset | DataSet1 |
|  | Filter | <none> |
|  | Weight | <none> |
|  | Split File | <none> |
|  | N of Rows in Working Data File | 1202 |
| Missing Value Handling | Definition of Missing | User-defined missing values are treated as missing. |
|  | Cases Used | Statistics are based on cases with no missing values for any variable used. |
| Syntax | | REGRESSION  /MISSING LISTWISE  /STATISTICS COEFF OUTS CI(95) BCOV R ANOVA COLLIN TOL CHANGE ZPP  /CRITERIA=PIN(.05) POUT(.10)  /NOORIGIN  /DEPENDENT Last_month_physical_activity_frequency  /METHOD=ENTER Age Home_digital_physical_activity_modalities. |
| Resources | Processor Time | 0:00:00.000 |
|  | Elapsed Time | 0:00:00.000 |
|  | Memory Required | 1812 bytes |
|  | Additional Memory Required for Residual Plots | 0 bytes |

| **Variables Entered/Removed** | | | |
| --- | --- | --- | --- |
| Model | Variables Entered | Variables Removed | Method |
| 1 | Home digital physical activity modalities, Age^a^ | . | Enter |
| a. All requested variables entered. | | | |

| **Model Summary** | | | | | | | | | |
| --- | --- | --- | --- | --- | --- | --- | --- | --- | --- |
| Model | R | R Square | Adjusted R Square | Std. Error of the Estimate | Change Statistics | | | | |
|  |  |  |  |  | R Square Change | F Change | df1 | df2 | Sig. F Change |
| 1 | .344^a^ | .100 | .100 | 1.27768 | .118 | 75.049 | 2 | 1121 | .000 |
| a. Predictors: (Constant), Home digital physical activity modalities, Age | | | | | | | | | |

| **ANOVA^b^** | | | | | | |
| --- | --- | --- | --- | --- | --- | --- |
| Model | | Sum of Squares | df | Mean Square | F | Sig. |
| 1 | Regression | 245.028 | 2 | 122.514 | 69.51 | .000^a^ |
|  | Residual | 1829.989 | 1121 | 1.632 |  |  |
|  | Total | 2075.018 | 1123 |  |  |  |
| a. Predictors: (Constant), Home digital physical activity modalities, Age | | | | | | |
| b. Dependent Variable: Last month physical activity frequency | | | | | | |

| **Coefficients^a^** | | | | | | | | | | | | | |
| --- | --- | --- | --- | --- | --- | --- | --- | --- | --- | --- | --- | --- | --- |
| Model | | Unstandardized Coefficients | | Standardized Coefficients | t | Sig. | 95.0% Confidence Interval for B | | Correlations | | | Collinearity Statistics | |
|  |  | B | Std. Error | Beta |  |  | Lower Bound | Upper Bound | Zero-order | Partial | Part | Tolerance | VIF |
| 1 | (Constant) | 1.400 | .145 |  | 8.347 | .000 | .927 | 1.497 |  |  |  |  |  |
|  | Age | .0007 | .002 | .127 | 3.42 | .000 | .006 | .016 | .119 | .134 | .127 | .999 | 1.001 |
|  | Home digital physical activity modalities | .260 | .022 | .323 | 11.36 | .000 | .224 | .316 | .319 | .325 | .323 | .999 | 1.001 |
| a. Dependent Variable: Last month physical activity frequency | | | | | | | | | | | | | |

| **Coefficient Correlations^a^** | | | | |
| --- | --- | --- | --- | --- |
| Model | | | Home digital physical activity modalities | Age |
| 1 | Correlations | Home digital physical activity modalities | 1.000 | .027 |
|  |  | Age | .027 | 1.000 |
|  | Covariances | Home digital physical activity modalities | .001 | 1.566E-6 |
|  |  | Age | 1.566E-6 | 5.993E-6 |
| a. Dependent Variable: Last month physical activity frequency | | | | |

| **Collinearity Diagnostics^a^** | | | | | | |
| --- | --- | --- | --- | --- | --- | --- |
| Model | Dimension | Eigenvalue | Condition Index | Variance Proportions | | |
|  |  |  |  | (Constant) | Age | Home digital physical activity modalities |
| 1 | 1 | 2.781 | 1.000 | .01 | .01 | .03 |
|  | 2 | .176 | 3.970 | .02 | .15 | .07 |
|  | 3 | .042 | 8.117 | .97 | .84 | .06 |
| a. Dependent Variable: Last month physical activity frequency | | | | | | |
