# Supplementary material for: The impact of COVID-19 lockdown on physical activity and weight gain among active adult population in Israel: a cross-sectional study
Source: BMC Public Health. 2021 Aug 6;21:1521. doi: 10.1186/s12889-021-11523-z (PMC8343341; doi:10.1186/s12889-021-11523-z)
Supplement: Supplementary file 4 — Additional file 4. ST4 Regression weight gain. [file 12889_2021_11523_MOESM4_ESM.docx]

**Regression weight gain**

**Regression**

| **Notes** | | |
| --- | --- | --- |
| Input | Data | D:\SharonDocuments\Desktop\Corona and PA.sav |
|  | Active Dataset | DataSet1 |
|  | Filter | <none> |
|  | Weight | <none> |
|  | Split File | <none> |
|  | N of Rows in Working Data File | 1202 |
| Missing Value Handling | Definition of Missing | User-defined missing values are treated as missing. |
|  | Cases Used | Statistics are based on cases with no missing values for any variable used. |
| Syntax | | REGRESSION  /MISSING LISTWISE  /STATISTICS COEFF OUTS CI(95) BCOV R ANOVA COLLIN TOL CHANGE ZPP  /CRITERIA=PIN(.05) POUT(.10)  /NOORIGIN  /DEPENDENT Weight gain  /METHOD=ENTER Age Last_month_physical_activity_frequency Home_digital_physical_activity_modalities. |
| Resources | Processor Time | 0:00:00.031 |
|  | Elapsed Time | 0:00:00.017 |
|  | Memory Required | 2108 bytes |
|  | Additional Memory Required for Residual Plots | 0 bytes |

| **Variables Entered/Removed** | | | |
| --- | --- | --- | --- |
| Model | Variables Entered | Variables Removed | Method |
| 1 | Home digital physical activity modalities, Age, Last month physical activity frequency^a^ | . | Enter |
| a. All requested variables entered. | | | |

| **Model Summary** | | | | | | | | | |
| --- | --- | --- | --- | --- | --- | --- | --- | --- | --- |
| Model | R | R Square | Adjusted R Square | Std. Error of the Estimate | Change Statistics | | | | |
|  |  |  |  |  | R Square Change | F Change | df1 | df2 | Sig. F Change |
| 1 | .399^a^ | .158 | .156 | 1.24712 | .159 | 69.700 | 3 | 1107 | .000 |
| a. Predictors: (Constant), Home digital physical activity modalities, Age, Last month physical activity frequency | | | | | | | | | |

| **ANOVA^b^** | | | | | | |
| --- | --- | --- | --- | --- | --- | --- |
| Model | | Sum of Squares | df | Mean Square | F | Sig. |
| 1 | Regression | 325.212 | 3 | 108.404 | 71.05 | .000^a^ |
|  | Residual | 1721.718 | 1107 | 1.555 |  |  |
|  | Total | 2046.931 | 1110 |  |  |  |
| a. Predictors: (Constant), Home digital physical activity modalities, Age, Last month physical activity frequency | | | | | | |

b. Dependent Variable: Weight gain

| Model | | Unstandardized Coefficients | | Standardized Coefficients | t | Sig. | 95.0% Confidence Interval for B | | Correlations | | |
| --- | --- | --- | --- | --- | --- | --- | --- | --- | --- | --- | --- |
|  |  | B | Std. Error | Beta |  |  | Lower Bound | Upper Bound | Zero-order | Partial | Part |
| 1 | (Constant) | 2.469 | .147 |  | 17.493 | .000 | 2.278 | 2.853 |  |  |  |
|  | Age | -.004 | .002 | -.071 | -1.949 | 0.051 | -.011 | -.001 | -.116 | -.077 | -.071 |
|  | Last month physical activity frequency | -.385 | .028 | -.378 | -13.357 | .000 | -.436 | -.320 | -.392 | -.361 | -.354 |
|  | Home digital physical activity modalities | -.008 | .024 | -.017 | -.353 | .723 | -.062 | .034 | -.138 | -.017 | -.016 |

**Coefficient Correlations^a^**

| Model | | | Home digital physical activity modalities | Age | Last month physical activity frequency |
| --- | --- | --- | --- | --- | --- |
| 1 | Correlations | Home digital physical activity modalities | 1.000 | .065 | -.331 |
|  |  | Age | .065 | 1.000 | -.133 |
|  |  | Last month physical activity frequency | -.331 | -.133 | 1.000 |
|  | Covariances | Home digital physical activity modalities | .001 | 3.871E-6 | .000 |
|  |  | Age | 3.871E-6 | 5.857E-6 | -9.438E-6 |
|  |  | Last month physical activity frequency | .000 | -9.438E-6 | .001 |

a. Dependent Variable: Weight gain

**Collinearity Diagnostics^a^**

| Model | Dimension | Eigenvalue | Condition Index | Variance Proportions | | | |
| --- | --- | --- | --- | --- | --- | --- | --- |
|  |  |  |  | (Constant) | Age | Last month physical activity frequency | Home digital physical activity modalities |
| 1 | 1 | 3.635 | 1.000 | .00 | .01 | .01 | .01 |
|  | 2 | .178 | 4.517 | .04 | .19 | .06 | .03 |
|  | 3 | .145 | 5.013 | .01 | .02 | .91 | .05 |
|  | 4 | .042 | 9.292 | .95 | .79 | .01 | .07 |

a. Dependent Variable: Weight gain
